# Phase Fraction Estimation in Multicomponent Alloy from EDS Measurement Data

**DOI:** 10.3390/ma17102322

**Published:** 2024-05-14

**Authors:** Andriy Burbelko, Tomasz Wiktor, Aldona Garbacz-Klempka, Eugeniusz Ziółkowski

**Affiliations:** Faculty of Foundry Engineering, AGH University of Krakow, 23 Reymonta Str., 30-059 Krakow, Poland; abur@agh.edu.pl (A.B.); agarbacz@agh.edu.pl (A.G.-K.); ez@agh.edu.pl (E.Z.)

**Keywords:** evaluation of phase fraction, X-ray microanalysis, QuantMap

## Abstract

To perform quality assessments of both metal alloys and many other engineering materials, measurements of the volume fractions of phases or microstructure components are utilized. For this purpose, quantitative analysis of the evaluated components’ distribution on metallographic specimens is often employed. Phases or components of the microstructure are identified based on the variation in signal received in the band of light seen. Problems with the correct identification of measurement results in this spectral band can be caused by the inhomogeneity of the etching when the alloy components are segregated. Additional uncertainty arises when the analyzed image pixel contains a boundary between grains of different phases. This article attempts to use the results of local chemical composition measurements as a source signal for quantitative evaluation of phase composition. For this purpose, quantitative maps of elemental concentration distributions, obtained with a Tescan Mira GMU high-resolution scanning electron microscope in QuantMap mode, were used as input data for the phase composition evaluation of an EN AC 46000 alloy sample. The X-ray microanalysis signal generation area may contain grains of more than one phase. Therefore, evaluation of the phase fractions in areas of individual measurements were calculated by looking for the minimum of the objective function, calculated as the sum of the squares of the deviations of the results of measurements of the concentration of individual elements from the weighted average values of solubilities of these elements in the phases.

## 1. Introduction

In the stereological evaluation of volume fraction in alloys according to Cavalieri’s principle, measurements are made on the surface of the metallographic test pieces using the linear or point method. According to this principle, in the case of the linear method, the volume fraction of a phase or microstructure component is equal to the relative length of the secant lines of the measurement system allocated to that phase. In the case of the point method, the volume fraction is determined as the relative number of corresponding points of the measurement grid [1,2]. In both cases, a tool is needed to identify the belonging of the analyzed points to a specific phase.

For non-automated quantitative evaluation of the microstructure image of alloys, both methods can be utilized. The linear method is more effective if, at the magnification used, the average section line size of the grains of the analyzed components is noticeably larger than the unit length of the measuring ruler. Otherwise, the method of counting the points of the measurement grid that are located in the area of the analyzed component is more convenient. Modern computer tools used to evaluate the stereological microstructure of alloys are actually based on the point method, analyzing individual pixels of the bitmap of the microstructure image.

The differentiation of microstructure components during analysis in the spectral range of seen light is based on the intensity and/or the color that the component takes on after a specially selected metallographic sample preparation. A variety of methods and reagents for etching are used to discriminate and identify the phase identity of the surface areas of metallographic specimens [3,4,5]. Nevertheless, during the analysis of solid solutions and fine-grained multiphase materials in the as-cast state, the assignment of a particular phase to some parts of the sample surface may be uncertain. This uncertainty may be due to the high spatial heterogeneity of the chemical composition and the corresponding appearance of the sample surface in solid solutions, or when the analyzed pixel hits an interfacial boundary in fine-grained materials.

For mapping phase fraction in extruded 6xxx aluminum alloys, correspondence between scanning electron microscopy (SEM) and visible light microscopy was first established for phase recognition in [6]. The established method of phase discrimination was then used in visual-light stereology measurement. On this basis, a point counting method was used to determine the volume fraction of phases, using a high number of metallographic sections at the lowest possible magnification.

A series of works have been reported in [7], in which electron backscatter diffraction (EBSD) maps were used to characterize the components of the steel’s microstructure. In the metallographic procedure presented in [7], visual-light microscopy was combined with EBSD mapping. This method was employed to evaluate the martensite fraction in dual phase steel after intercritical annealing and isothermal treatment.

A situation of uncertainty occurs when analyzing the phase composition of alloys in thin-walled castings made from alloys based on the Al-Si system used in die casting. These alloys have a multiphase microstructure. The objectivity of the identification can be increased using the results of chemical composition measurements from SEM using energy-dispersive X-ray microanalysis (EDS) for primary data acquisition instead of the image taken in the visible light spectrum. The results of measuring the local chemical composition are objective and do not depend on user decisions.

The combination of SEM and EDS provides extensive opportunities for quantitative evaluation of the microstructural features of a variety of materials. The possibilities for identifying measurement results are more advanced in this case. The report of [8] presents an example of quantitative analysis of the phase composition of 94ND10 material based on high-contrast maps obtained by EDS. The evaluated material is a mixture of oxides Al_2_O_3_, MgO, CaO, and SiO_2_. The maps distinguish areas of Al-rich, Mg-rich, and Ca/Si-rich phases. It was noted that the Mg-rich phase contains Al. Nevertheless, the large difference contrasting the concentration distribution map obtained by Energy Dispersive Spectroscopy (EDS) between the Al-rich and Mg-rich phases allows their unambiguous identification by adjusting the contrast threshold accordingly.

In [9,10], a combination of scanning electron microscopy with X-ray microanalysis and image processing was used to quantify the phase composition of ceramic materials. Each of these techniques can provide some information not available from the other. Thus, a quantitative characterization of microstructural features in these materials was carried out.

This article presents a method for quantitative analysis of the phase composition of an alloy used to make high-pressure die castings (HPDCs) based on the results of local chemical composition by means of EDS measurements. In alloys of this type, the interpretation of the measurement results is not as unambiguous as in the case of the oxide mixture described above [8,9,10]. In such studies, it is not always possible to obtain a high visual contrast between the grain surfaces of the different phases, so direct interpretation of the results is not always feasible. Additional uncertainty in the results obtained on the base of visible light spectrum is related to the high density of interfacial boundaries, measured as the ratio of the area of the grain boundaries to the volume of the area containing them. The use of the results of measurements of local chemical composition obtained by X-ray microanalysis as source data for quantitative evaluation of phase composition eliminates the aforementioned uncertainties.

The possibility of using the results of EDS microanalysis to quantify the phase composition of an alloy may also be attractive for the fact that this measurement method is considered to be relatively fast.

## 2. Materials and Methods

An aluminum-based alloy EH AC-46000, which is commonly used for HPDC, was selected for this study. Samples taken from a test HPDC of this alloy were used for this study in the as-received state without additional heat treatment. The actual chemical composition of the alloy is given in Table 1. In terms of silicon and other components, this chemical composition corresponds to the current standard [11]. Measurements of the elemental composition of the analyzed sample were made by a SPECTROMAXx spark spectrometer from SPECTRO Analytical Instruments GmbH (Kleve, Germany).

**Figure 1:**
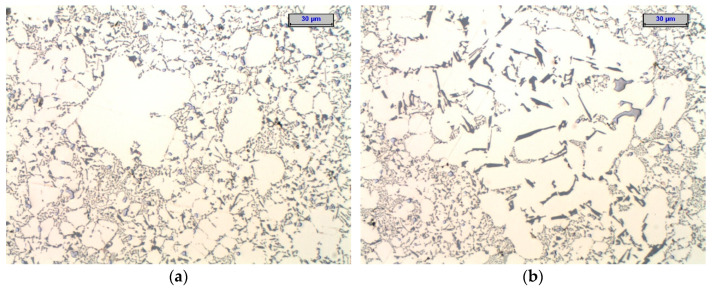
Example of microstructure heterogeneity of EN AC 46000 alloy in die casting walls: (**a**) near the mold wall; (**b**) in the hot spot of casting.

Solidification in HPDC metal die occurs under conditions of intensive cooling and high rates of temperature change. As a result, the phase composition of the alloy in such a casting may differ from the equilibrium state. In addition, the local microstructure and phase composition of the alloy varies spatially depending on the thickness of the casting wall and the distance from the die surface. This is because they depend on the local intensity of heat removal, and therefore the rate of temperature change. The variation in micro-structure results in heterogeneity of mechanical and functional properties. In addition, the microstructure of thin-walled die castings (an example is shown in Figure 1) is distinguished by small grain sizes, which also complicates its stereological quantitative assessment with the use of visible light.

## 3. Example of Quantitative Analysis in the Visible Light Band

The subject of the elementary act of measurement in stereological quantitative analysis is, in reality, a small area of finite dimensions (a pixel), not a dimensionless point on the surface of the metallographic specimen. This holds true regardless of whether a recorded computer bitmap of the image is analyzed or whether the analysis is performed directly from the original microstructure image by an operator without computer assistance. The size of such a pixel is determined by the magnification used and the resolution provided by the microscope used for the measurements. The inhomogeneity of the solid solution and the high density of interfacial boundaries for a fine-grained structure similar to that shown in Figure 1 mean that the assignment of a particular phase to an analyzed image pixel can be uncertain.

**Figure 2:**
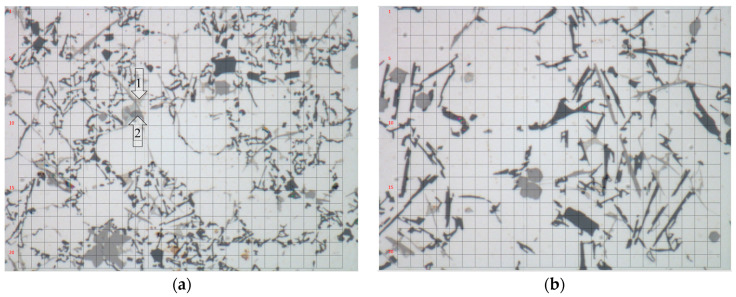
Measuring grid of the point method for evaluating phase contributions in EN AC 46000 alloy castings: (**a**) near the mold wall (arrows 1 and 2 indicate non-metallic phases 1 and 2, Table 2); (**b**) in the hot spot of casting.

The point method measurements in the visible light spectrum presented below were carried out on samples taken from areas near the casting surface and in the center of the casting wall. The actual assignment of an uncertain pixel of an image, as shown in Figure 2, in visible light microscopy to a specific phase depends on several factors, including the inhomogeneity of grain surface coloration within the same phase and the variation in grain coloration across different phases. Uncertainty is very high when the pixel partially or fully overlaps the width of the grain boundary in the image. In such cases, the accuracy of the assessment may rely on the individual experience of the person conducting the measurement. Uncertainty is also challenging to estimate for automatic measurements.

The number of grid measurement points shown in Figure 2 is 546. The results obtained for the six measurement fields for each sample, yielding a total of *N* = 3276 points, are presented in Table 2. The mathematical apparatus of binomial statistical decomposition can be used to evaluate the results of measuring the volume fractions of individual microstructure components using the point method. For this statistical distribution, the standard deviation *S_n_* of the number of measurement points *n* corresponding to the selected phase is calculated as follows:

(1) $$S_{n}=\sqrt{N\cdot p\cdot \left(1-p\right)},$$

where *p* is the probability of occurrence of this phase. It is equal to the calculated fraction of a given phase.

The expected values of the volume shares of each phase *f* and their standard deviations *S_f_* were calculated by dividing the values of *n* and *S_n_* by the number of measurement points *N*

(2) $$f=\frac{n}{N},$$

(3) $$S_{f}=\frac{S_{n}}{N}.$$

The results of the measurements with calculated values of evaluation are shown in Table 2.

## 4. EDS Measurement Data Acquisition Method

Microanalysis of the local chemical composition of the sample was carried out by SEM using a high-resolution Tescan Mira GMU microscope with an FEG electron source. The topography of the sample was examined using solid-state detectors at a beam energy of 18 keV. The chemical composition was analyzed by EDS using an Ultim Max EDS detector from Oxford Instruments (Abingdon, UK). AZtec^®^ 6.0. SP1 (Oxford Instruments software) was used to process the EDS signal.

The use of EDS measurements for quantification of the spatial distribution of lithium using backscattered electron microscopy measurements is described in [12]. A Tescan Mira 3 field-emission scanning electron microscope (TESCAN GROUP, a.s., Brno, Czech Republic) was used for this purpose. The need for additional signal registration of backscattered electrons (BE) in this case is related to the small atomic number of lithium (Z_Li_ = 3). Determining the content of an element with such a small atomic number is a difficult challenge for EDS spectroscopy. Therefore, in the cited article, the BE signal was also used for quantification purposes. The software toolkit of the microscope for quantification of EDS measurement results applies corrections based on atomic number (Z), absorbency (A), and fluorescence (F) correction factors (known as ZAF corrections).

In the alloy analyzed in presented paper, the smallest atomic number was that of Mg (*Z*_Mg_ = 12). The determination of magnesium and other elements with higher atomic numbers is not fraught with such difficulties. The results of a reference-free measurement often used in high-speed EDS measurements were used for further processing.

Available mapping modes of the Aztec^®^ software used include the standard Window Integral Maps (also known as raw X-ray maps without correction) and TruMaps (maps that are corrected for background and peak overlap) [13]. Additionally, the QuantMap mode utilized in this study facilitates the acquisition of quantitative elemental maps, enabling visualization of the spatial distribution of elemental concentrations within a sample. In QuantMap mode, maps are generated based on spectral data collected from each pixel, which are then deconvolved and corrected.

**Figure 3:**
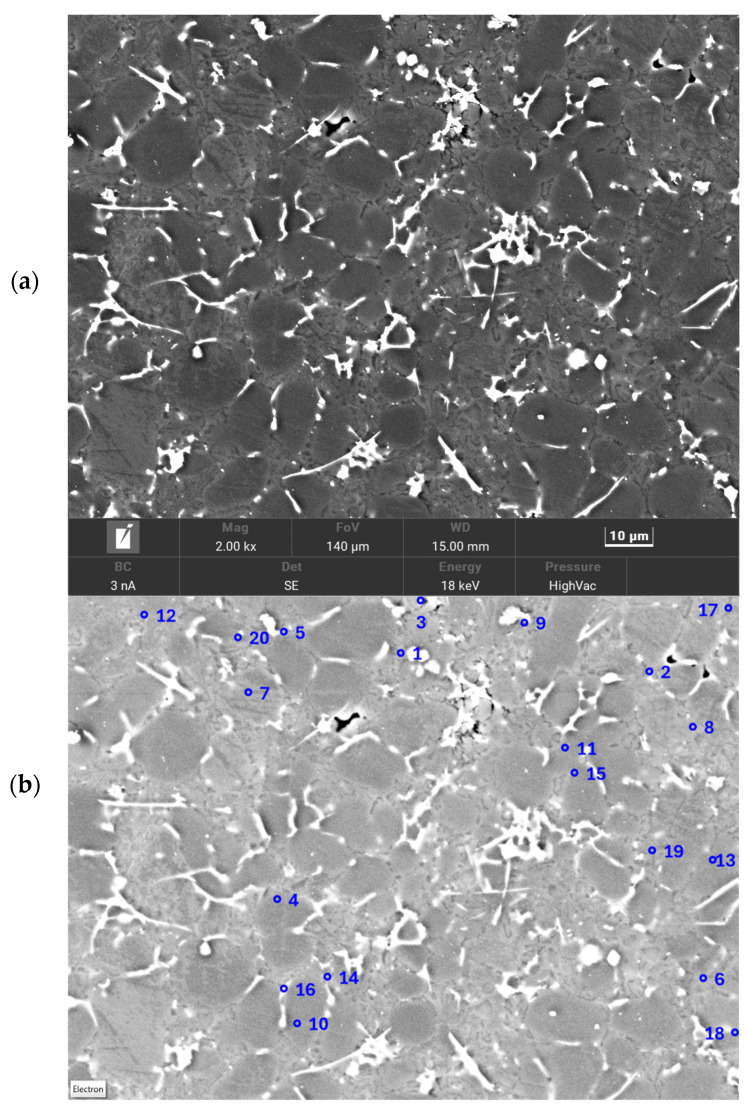
Microstructure of EN AC 46000 alloy in HPDC sample (SEM) (**a**) and the microanalysis area of the chemical composition with the highlighted points described in the Table 3 (**b**).

**Figure 4:**
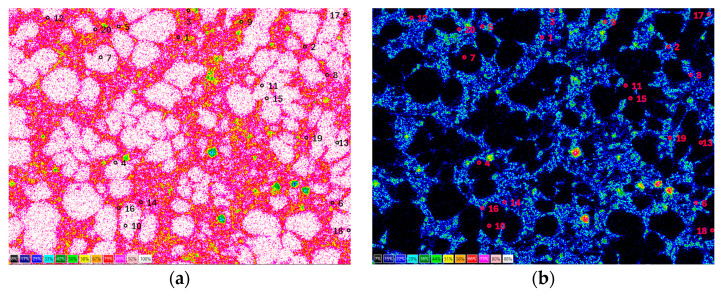

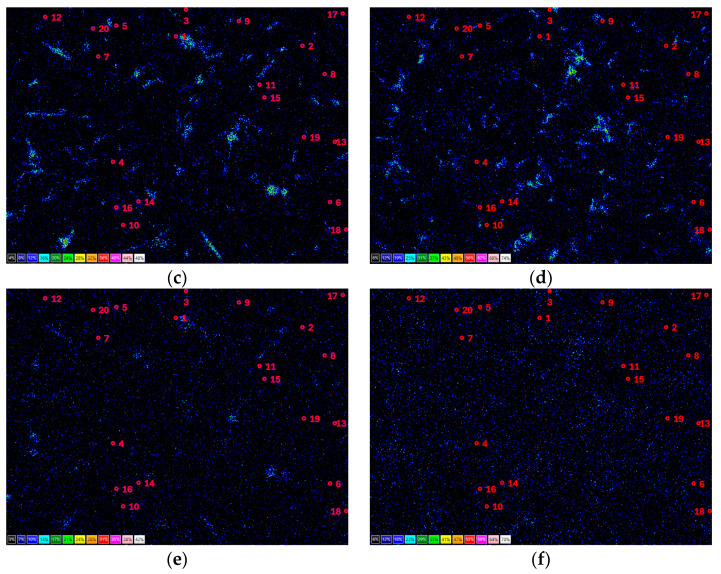
QuantMaps of the content of selected components of EN AC 46000 alloy [wt.%], obtained on the grid of 512 × 384 points for the sample area shown in Figure 3b: (**a**) Al, (**b**) Si, (**c**) Fe, (**d**) Cu, (**e**) Mn, and (**f**) Zn, with the highlighted points described in the Table 3.

An example of QuantMap component concentration maps for the EN AC 46000 alloy sample area, as depicted in Figure 3b, is provided in Figure 4. The results presented here cover an area of 140 × 105 µm and were obtained on an analytical grid of 512 × 384 points. Measurements were performed for a Pixel Dwell Time of 50 µs per measurement point. The analyzed area was scanned 18 times to increase measurement accuracy.

Given the advantageous ratio of data quantity to measurement execution time, an effort was made to utilize the results obtained in QuantMap mode for the quantitative evaluation of the microstructure of the analyzed alloy sample.

**Figure 5:**
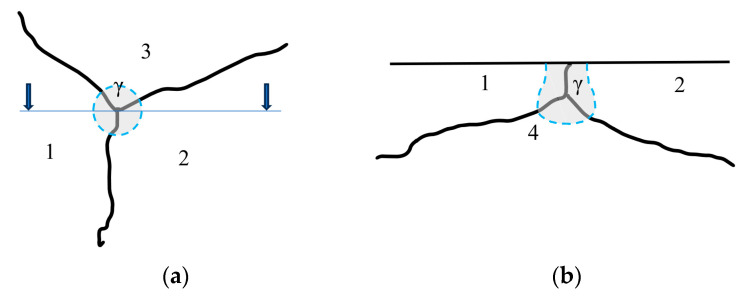
Generation zone of characteristic X-ray involving grains of 4 phases (1–4): (**a**) view normal to the surface of the sample analyzed; (**b**) schematic of the cross-section of the sample through the point of contact of three grains on the metallographic specimen (in the position indicated by arrows in (**a**)); γ—area of X-ray radiation generation; 1–4—grains of different phases.

In contrast to visible light microscopy, the generation area of characteristic X-rays is three-dimensional, extending into the analyzed sample to a depth of approximately 2 µm [14]. Therefore, we are dealing with ‘voxels’ rather than ‘pixels’. The generation area for characteristic X-ray radiation can encompass grains from more than three phases, as demonstrated in Figure 5. The determined contents of individual elements in such a measurement should reflect the weighted average of their concentrations in the grains of the phases within the measurement area, with weights proportional to the volume contributions of these grains.

The potential absence of high contrast between the signals from different phases, the inhomogeneity of the chemical composition, and the scenario where several grains of different phases may be included in the generation area of characteristic X-ray radiation necessitate a specialized approach for analyzing EDS measurement results. Directly determining the phase composition at a single datapoint by solving a system of algebraic equations is unfeasible under these circumstances. The chemical composition’s heterogeneity across different phases and the characteristic radiation generation from a voxel containing more than one phase compels the adoption of optimization methods [15,16,17].

## 5. Determination of Phase Composition from the Results of EDS Measurements

In Al-Si-based alloys used in the HPDC process, in addition to the basic microstructure components such as α-Al-based FCC solid solution grains and silicon grains, depending on the concentration of elements such as Fe, Cu, Mn, and impurities (e.g., Cr, Ni), various intermetallic phases can be present. An analysis of the effects of these components on the microstructure of the alloys and on the type of intermetallics found is presented in other publications [18,19,20,21]. Grains of solid solutions and some intermetallic phases formed under conditions of rapid crystallization in die casting have a heterogeneous chemical composition.

Data on the possibility of occurrence of the various phases and the assessment of the solubility limits of the components further applied to the calculations were obtained using the CompuTherm thermodynamic database in the ProCAST 2023.0 software suite, ESI Group^®^ [22]. The range of change in the equilibrium solubility limits of the components in the phases of EN AC 46000 alloy during equilibrium crystallization between liquidus and solidus temperatures is shown in the Table 4.

In the general case for the evaluation of mass fractions of *P* phases based on the measurement of the local chemical composition of *M* elements (for M≥ *P*), it is proposed to find the minimum of the objective function *F* calculated as the sum of the squares of the deviations of the results of measurements of the concentration of individual elements (*C*) from the weighted average values predicted by the thermodynamic database, detailed in the Table 4:

(4) $$F=\sum_{i=1}^{M}\left\{w_{i}\cdot {\left[C_{i}-\sum_{j=1}^{P}\left(\frac{B_{i,j}^{\min}+B_{i,j}^{\max}}{2}\cdot x_{j}\right)\right]}^{2}\right\}\to \min,$$

where:

*x_j_*—the evaluated value of the fraction of the *j*-th phase, wt. %;

*C_i_*—the measurement result of the content of the *i*-th chemical element in the analyzed point, wt. %;

$B_{i,j}^{\min},\;B_{i,j}^{\max}$—lower and upper solubility limits, respectively, of the *i*-th chemical element in the *j*-th phase, wt. % (see Table 4);

*w_i_*—the weighting factor for the deviation of the content of element *i* in the analyzed point (described below).

A step-by-step algorithm diagram, which shows the calculation path on the basis of Equation (4), is presented in the Supplementary Materials.

In the case of X-ray microanalysis of the concentration of alloying elements, the value of the evaluated content depends on the difference between the height of the characteristic X-ray peak of the analyzed element and the background level. The intensity of these signals is measured by the number of X-ray photons detected during the measurements made for each point of the analyzed area.

**Figure 6:**
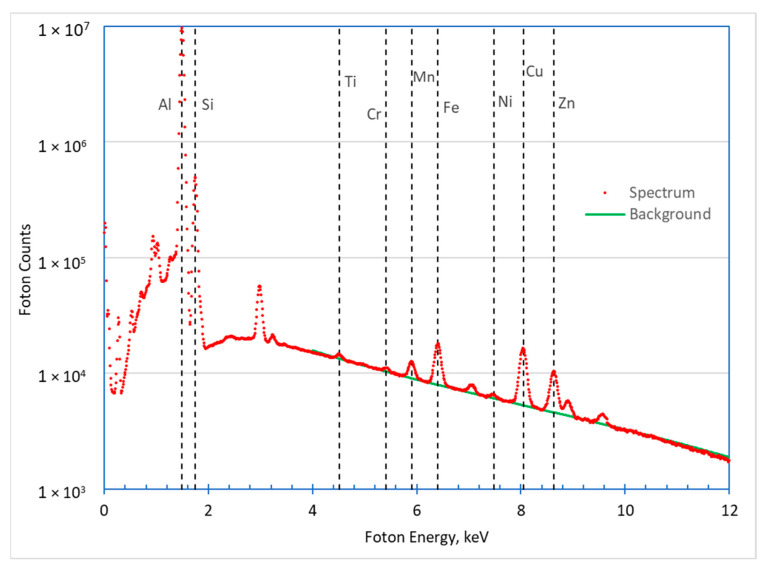
X-ray spectra from the sample area (dots), the approximation of the background signal (solid line), and the position of the characteristic K_α_ spectral lines of the analyzed elements (vertical dashed lines).

Spectrum results for the sample area shown in Figure 3 are shown in Figure 6. In this figure, the positions of the characteristic K_α_ spectral lines of the elements are marked, based on which the contents of the alloy components were calculated for QuantMap (Figure 4). The approximation of the background signal is also shown for photon energies in the part of the spectrum above 4 keV. The spectral background distribution in this figure is approximated by the Equation of type [14],

(5) $$G=K\frac{E_{0}-E}{E},$$

where *G* is the number of detected photons with energy *E* [keV] and *E*_0_ and *K* are constants.

The values of the parameters of this equation (*E*_0_ = 16.47 keV and *K* = 5060) were determined by the least-squares method based on areas of the spectrum outside the range of characteristic peaks.

The behavior of such signals follows a Poisson statistical distribution. For this statistical distribution, the value of the standard deviation of both the intensity of the characteristic signal and the background level are equal to the square roots of these intensities. It follows that the uncertainty of concentration estimation increases with a decrease in the “signal:background” ratio, and thus increases with a decrease in the component content.

The objective function in the mathematical model represented by Equation (4) is the sum of the squares of the deviations of the content of individual chemical elements from the center of the predicted ranges of their concentration. In order to balance the importance of different elements in the objective function, it is necessary to introduce weighting factors *w_i_*. This is due to the fact that measurements of the concentration of individual elements in X-ray microanalysis have varying uncertainties. For elements with smaller uncertainties, a smaller value of the weighting factor should be used. Thus, the impact of the deviation of the concentration estimate from the center of the prediction interval for measurements with lower uncertainty will have less impact on the change in the objective function than such a deviation predicted for elements with higher measurement uncertainty.

QuantMap data acquisition does not have peak and background intensity information for local signals. Therefore, within the framework of the presented method, the values of the weight coefficients in Equation (5) for element *i* at measurement point *k* were determined on the basis of the concentration of the element whose content at this measurement point was the highest ($C_{k}^{\max}$) according to the formula

(6) $$w_{i,k}=\frac{C_{k}^{\max}}{C_{i,k}}.$$

In addition, constraints are applied for the searched values of phase fractions:

(7) $$\left\{\begin{array}{l} \sum_{j=1}^{P}x_{j}=100\%\\ x_{j}\geq 0,\;j=1,2,\ldots \;P \end{array}\right.$$

The objective function in this analysis is modeled as a quadratic function, while the constraints are represented by linear functions. Thus, the determination of the phase proportions can be achieved by numerically solving a quadratic programming problem, which is a specific instance of a nonlinear mathematical programming problem [23].

Calculations to evaluate the phase composition were conducted based on results from 20 points derived from the QuantMap quantitative measurements. These points were selected where the aluminum (Al) content was below the lower solubility limit of this element in the Face-Centered Cubic (FCC) Al-based solid solution, and the silicon (Si) content was below the lower solubility limit of this element in the Diamond phase. The concentration data for the components at these analyzed points after normalization are presented in Table 3.

**Figure 7:**
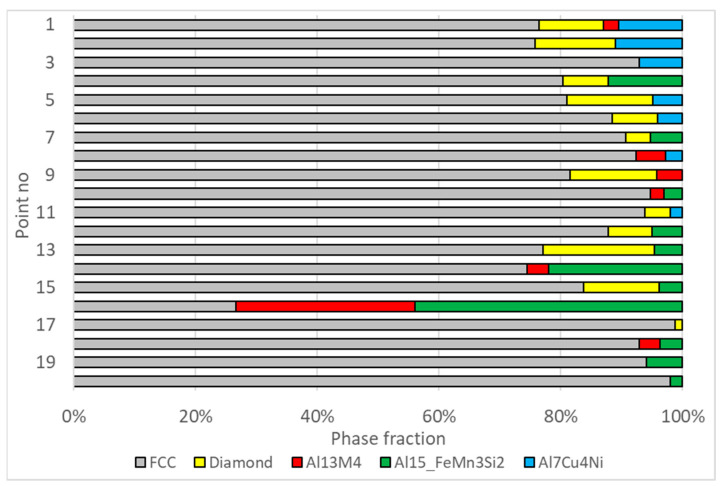
The results of the phase composition assessment for the measurement points summarized in Table 3.

The results of the phase composition evaluation, derived from EDS data, are depicted in Figure 7. As illustrated in the Figure, the β-AlFeSi phase, which was considered in the calculation assumptions, was not detected at the analyzed points. Alloy solidification under the condition of the HPDC process proceeds rapidly. Under these conditions, significant deviations of the solidification pass and the phase composition of the alloy from that predicted by the equilibrium thermodynamic system are possible. This is in agreement with the results of the occurrence of iron-rich intermetallics obtained when studying the phase composition of A383 aluminum alloy in HPDC [24]. In this article, the occurrence of phase β-AlFeSi was also not detected.

According to the evaluation, four measurement areas—specifically points 3, 17, 19, and 20—contained grains from two phases. At one measurement area (point 1), the presence of four phases was identified. The results for the remaining measurement areas indicate the inclusion of grains from three phases.

**Figure 8:**
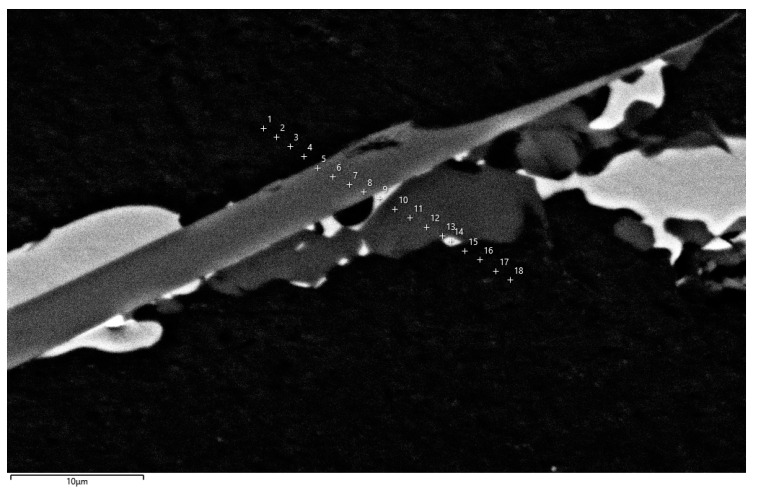
Positions and labels of points selected for microanalysis in sample obtained with low cooling rate.

In order to assess the reliability of the proposed method, phase fractions were measured in the sample of the analyzed alloy cooled over a range of crystallization temperatures at a rate of 0.1 K/s. The 18 measurement points shown in Figure 8 were selected for analysis. The results of the chemical composition microanalysis are shown in Table 5.

**Figure 9:**
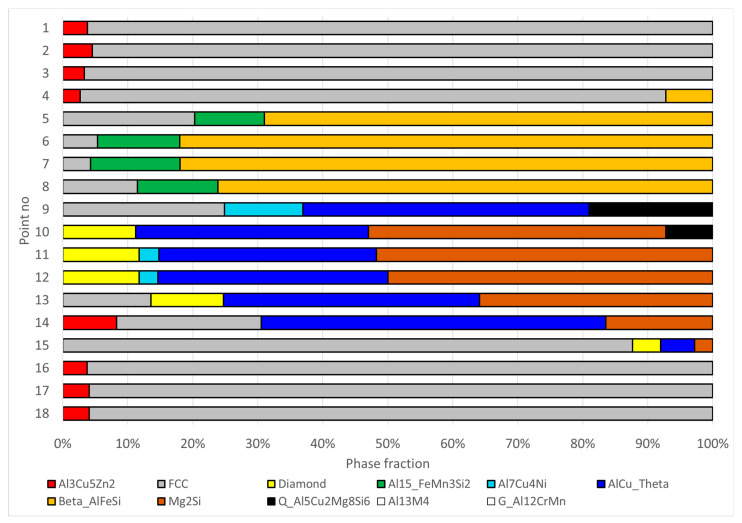
Phase composition calculated for points shown in Figure 8.

Based on the obtained results, an estimation of phase fraction was performed for these points. The results of the phase composition evaluation for this data set are shown in Figure 9. In the calculations, it was taken into account that during the slow cooling of the alloy samples the solid phase transformations will be observed. Therefore, the set of phases considered in the calculations (Table 4) was expanded. The additional phases considered in the optimization analysis and their chemical composition or solubility limits are listed in Table 6.

The obtained evaluation indicates that the analyzed points do not contain grains of phases designated as Al13M4 and G_AL12CrMn. These results also indicate the presence of another phase in the metal matrix; namely, at points 1–4, 14, and 16–18 there are Al3Cu5Zn2 grains in the amount of about 3–4%. This should not be taken as an unreliability of the proposed evaluation method. This phase is a product of solid–solid phase transformations occurring at temperature below 279 °C. Its equilibrium content at ambient temperature is 3.45%, which indirectly confirms the correctness of the proposed method.

**Figure 10:**
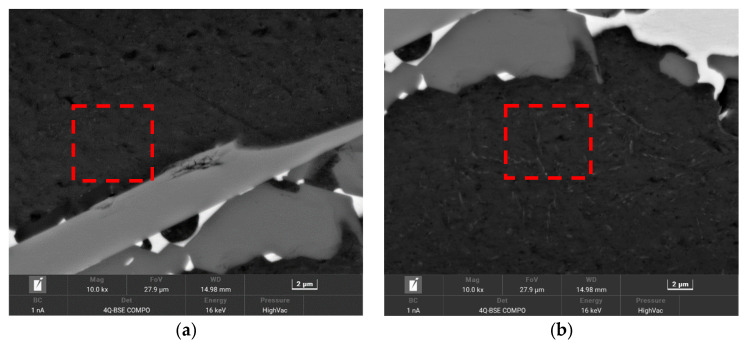
Heterogeneity of microstructure of Al-based solid solution in areas surrounding measurement points shown in Figure 8; marked area of points: (**a**) 1–3; (**b**) 15–18.

Using higher magnification (Figure 10) provide support for the presence of precipitates in the Al-based solid solution. However, the sizes of these precipitates are too small to determine their chemical composition by X-ray microanalysis.

This result indicates the potential applicability of the proposed method for evaluating multiphase microstructure components when the dimensions of the precipitates and the distances between them are sufficiently small to preclude metallographic evaluation using the visible light spectrum.

## 6. Discussion and Proposals for Further Research

The technique introduced in this article for the quantitative evaluation of the phase composition of the as-cast EN AC 46000 multiphase alloy, along with the preliminary results, suggests the potential applicability of local chemical composition measurements using EDS (specifically, the Tescan QuantMap type) for quantitative analysis of alloy microstructure. This method represents a notable advancement over the traditional stereological method of microstructure evaluation, which relies on visible light bands. The EDS approach offers greater objectivity, eliminating the relatively subjective or uncertain identification associated with measurements involving grains of multiple phases.

Contrary to metallographic analysis conducted with images obtained in the visible light spectrum, it is feasible to detect up to four phases simultaneously within a single measurement area when using characteristic X-ray evaluation. This capability stems from the three-dimensional nature of the characteristic X-ray generation area used in the analysis, which spans approximately 1–2 µm.

The proposed method necessitates an initial estimation of the solubility limits of alloy components within the phases under investigation. For this purpose, data from a thermodynamic database were utilized. Future research should explore the feasibility of further refining the assessment of the chemical composition of individual phases through measurements conducted by X-ray microanalysis.

Evaluations of the proportions of various phases at the measurement points were derived from the numerical solution of a quadratic programming task, identified as a specific instance of a nonlinear mathematical programming problem. In these evaluations, weighting factors—determined based on a preliminary assessment of the uncertainty associated with chemical composition measurements by the EDS method—were employed. Future studies should explore the method for optimizing the value of these weighting factors, considering the measurement conditions. This approach should incorporate both data on the local concentrations of components and the intensity levels of peak and background for the characteristic X-ray bands of the individual elements.

## Figures and Tables

**Table 1:** Chemical composition of tested alloy (Wt.%).

Components	Si	Fe	Cu	Mg	Cr	Ni	Zn	Pb	Sn	Ti	Mn	Al
Measured	9.64	0.89	2.23	0.30	0.04	0.07	1.1	0.09	0.03	0.05	0.28	Remainder

**Table 2:** Measurement results for the samples shown in Figure 2.

Phases	Near the Mold Wall				Middle of the Section			
	Points		Vol. Fraction, %		Points		Vol. Fraction, %	
	*n*	*S_n_*	*f*	*S_f_*	*n*	*S_n_*	*f*	*S_f_*
α-Al	2672	22.2	81.56%	0.68%	2810	20.0	85.78%	0.61%
Silicon	381	18.3	11.63%	0.56%	327	17.2	9.98%	0.52%
Non-metallic 1	141	11.6	4.30%	0.35%	66	8.0	2.01%	0.25%
Non-metallic 2	81	8.9	2.47%	0.27%	60	7.7	1.83%	0.23%
Microporosity	1	1.0	3 × 10^−4^	5 × 10^−6^	13	3.6	0.40%	0.11%

**Table 3:** Results of EDS measurements of alloy component concentrations at selected points [wt.%].

Point No.	Al	Cr	Cu	Fe	Mg	Mn	Ni	Si	Zn
1	67.28	0.845	16.51	0.590	1.824	1.089	0.272	9.560	1.681
2	75.46	0.617	6.198	1.073	0.949	0.725	0.613	11.74	2.483
3	87.59	0.878	4.221	0.169	0.791	0.543	1.077	2.483	2.048
4	79.67	0.306	3.270	3.190	0.758	1.133	1.034	8.807	1.722
5	75.95	0.399	2.838	0.468	2.471	0.577	1.132	13.95	1.500
6	83.28	0.878	2.541	0.725	0.508	0.453	1.420	7.774	2.300
7	87.42	0.927	2.351	0.728	1.047	0.898	0.485	5.107	0.615
8	90.83	0.724	2.142	1.386	0.243	0.449	0.623	1.026	1.801
9	77.04	0.575	1.977	1.173	1.770	0.341	1.892	14.09	0.910
10	86.59	1.067	1.871	1.000	2.003	0.349	0.168	1.938	4.438
11	88.72	0.737	1.751	0.549	0.935	0.357	0.630	4.641	1.283
12	85.31	0.646	1.650	0.817	0.564	0.673	0.669	8.162	0.963
13	74.39	0.117	1.534	0.798	1.268	0.586	0.148	18.85	1.924
14	80.10	0.293	1.407	4.302	1.593	2.195	1.298	5.315	2.924
15	78.20	0.592	1.269	0.574	1.406	0.543	1.249	12.74	3.238
16	65.96	2.370	1.173	15.80	0.458	5.197	1.458	6.443	0.929
17	91.18	0.446	1.058	0.085	1.563	0.499	1.628	1.827	1.145
18	90.85	0.812	0.910	1.451	0.140	0.451	0.950	1.732	2.368
19	91.26	0.404	0.718	1.187	1.109	0.597	1.217	1.408	1.357
20	93.63	0.565	0.438	0.208	0.834	0.729	0.612	0.924	1.631

**Table 4:** Solubility limits of components in the basic phases of EN AC 46000 alloy in the equilibrium crystallization temperature range [wt.%].

Phases		Al	Cr	Cu	Fe	Mg	Mn	Ni	Si	Zn
Al13M4	min	61.4	5.9	*	31.6	0	*	*	*	*
	max	61.4	6.9	*	32.7	0	*	*	*	*
Al15_FeMn3Si2	min	58.6	0	0	14.9	0	12.4	0	11.0	0
	max	59.8	0	0	17.7	0	15.1	0	11.3	0
Al7Cu4Ni		37.6	0	57.0	0	0	0	11.7	0	0
β-AlFeSi	min	54.5	0	0	26.8	0	*	*	15.7	0
	max	57.4	0	0	26.9	0	1	*	18.7	0
Diamond (Si)	min	*	0	0	0	0	0	0	99.9	*
	max	*	0	0	0	0	0	0	100.0	0.1
FCC (Al-base solid solution)	min	95.1	*	*	*	*	*	*	*	0.4
	max	99.3	*	2.2	*	3	0	0	1.4	1.3

* Concentration less than 0.01 wt.%.

**Table 5:** EDS measurement results for points indicated in Figure 8.

Label	Al	Cr	Cu	Fe	Mg	Mn	Ni	Si	Zn
1	92.22	0.0	2.70	0.11	0.22	0.10	0.07	0.78	3.79
2	92.09	0.0	3.06	0.23	0.12	0.0	0.05	0.63	3.83
3	92.49	0.04	2.46	0.27	0.12	0.0	0.0	0.84	3.78
4	90.58	0.0	2.01	1.77	0.01	0.06	0.15	1.83	3.59
5	62.58	0.05	0.63	20.12	0.02	1.51	0.60	13.29	1.21
6	56.53	0.0	0.89	23.93	0.0	1.80	0.34	15.99	0.53
7	56.43	0.0	0.50	24.15	0.0	1.96	0.54	16.16	0.25
8	59.56	0.0	1.03	21.67	0.14	1.72	0.39	14.77	0.72
9	51.09	0.0	32.81	2.17	4.65	0.20	1.36	7.05	0.67
10	18.12	0.0	20.39	0.27	30.99	0.14	0.37	29.66	0.06
11	15.78	0.01	20.31	0.20	32.63	0.0	0.34	30.54	0.19
12	17.98	0.0	19.73	0.18	31.51	0.18	0.34	29.90	0.19
13	31.13	0.03	21.15	0.19	22.63	0.0	0.15	24.26	0.46
14	47.53	0.02	32.44	0.24	8.41	0.06	0.40	9.56	1.35
15	85.86	0.01	3.72	0.05	1.89	0.03	0.34	5.89	2.21
16	92.56	0.01	2.79	0.15	0.41	0.11	0.20	1.27	2.49
17	93.49	0.05	2.98	0.0	0.12	0.0	0.03	0.77	2.56
18	93.74	0.0	2.99	0.0	0.07	0.12	0.0	0.63	2.45

**Table 6:** Solubility of components in additional intermetallic phases (Computherm).

Phases		Al	Cr	Cu	Fe	Mg	Mn	Ni	Si	Zn
Al3Cu5Zn2		29.7	0	55.9	0	0	0	0	0	14.4
AlCu_Theta	min	46.1	0	53.1	0	0	0	0	0	0
	max	46.9	0	53.9	0	0	0	0	0	0
G_Al12CrMn	min	85.7	3.30	0	0	0	11.0	0	0	0
	max	85.7	3.34	0	0	0	11.0	0	0	0
Mg2Si		0	0	0	0	0	0	0	36.6	0
Q_Al5Cu2Mg8Si6		21.6	0	20.3	0	31.1	0	0	27.0	0

## Data Availability

The raw data supporting the conclusions of this article will be made available by the authors on request.

## Supplementary Materials

The following supporting information can be downloaded at: https://www.mdpi.com/article/10.3390/ma17102322/s1, Figure S1: Flowchart of the evaluation algorithm used.
