# Supplementary material for: Phase Fraction Estimation in Multicomponent Alloy from EDS Measurement Data
Source: Materials (Basel). 2024 May 14;17(10):2322. doi: 10.3390/ma17102322 (PMC11122778; doi:10.3390/ma17102322)
Supplement: Supplementary file 1 [file materials-17-02322-s001.zip › materials-2926249-supplementary.pdf]

Stepwise algorithm chart indicating calculations followed for each measurement point selected

Flowchart of the evaluation algorithm used is presented in Figure S1.

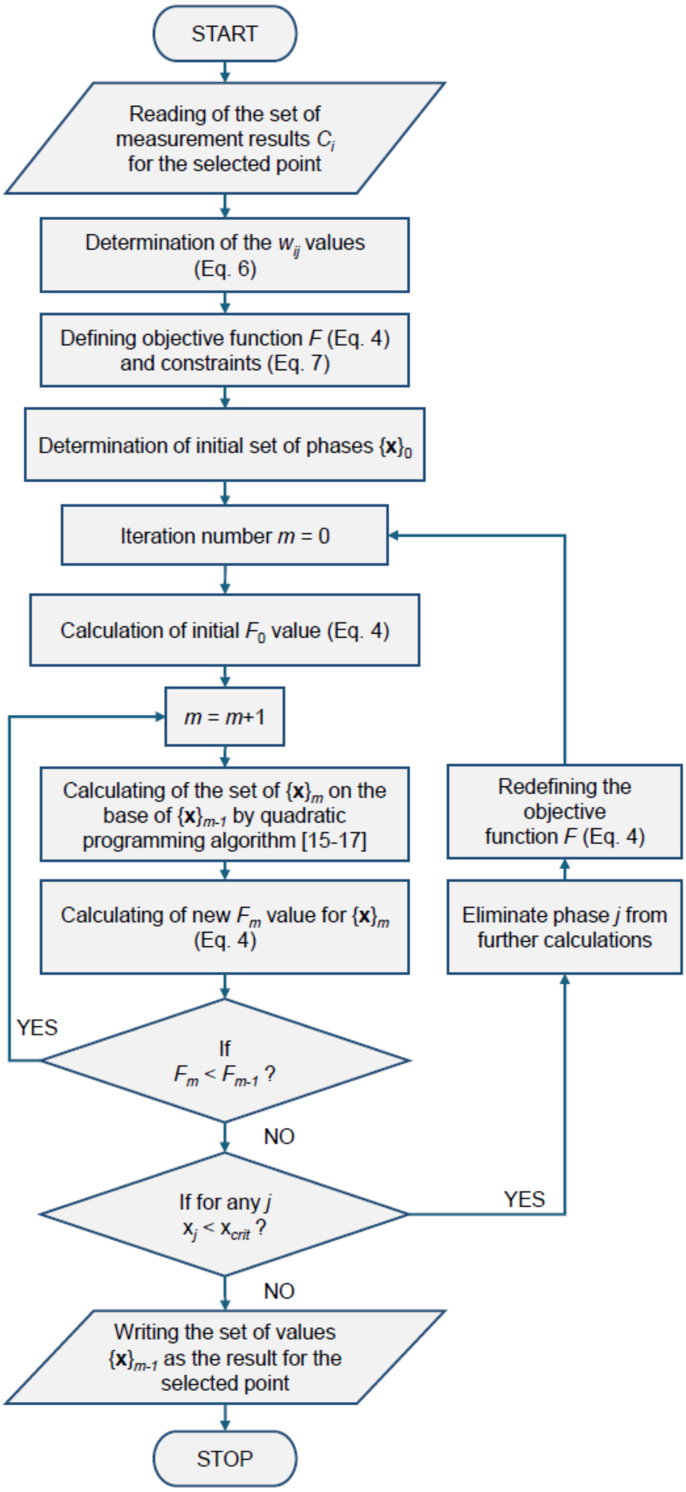

Figure S1. Flowchart of the evaluation algorithm used

For a set of analyzed phases and information about their chemical composition, an objective function F is created (Equation 4). For an arbitrary initial assumption about the proportions of the phases (x_j) on the basis of the results of measurements by means of quadratic programming algorithm [15-17], a vector $\{\mathbf{x}\}=(x_1,x_2...)$ is sought for which the function F has a minimum.

If any of the components of the resulting vector $\{\mathbf{x}\}$ is less than the critical value, these phases are eliminated from further evaluation. It means, that the evaluation of its content in the analyzed point is equal to 0.

A search for the F-function for the reduced set of phases is then performed. The evaluation is repeated until the content of each of the individual phases evaluated is greater than the critical value. The results presented in Figure 9 were obtained for the critical value of x_{crit} = 2%.
